# Resistive switching characteristics of carbon nitride supported manganese oxysulfide: an evidence for the sweep dependent transformation of polarity

**DOI:** 10.1038/s41598-020-71313-2

**Published:** 2020-08-31

**Authors:** Venkata K. Perla, Sarit K. Ghosh, Kaushik Mallick

**Affiliations:** Department of Chemical Sciences, University of Johannesburg, P.O. Box: 524, Auckland Park, 2006 South Africa

**Keywords:** Materials science, Nanoscience and technology

## Abstract

As part of a program to investigate the materials for resistive random access memory (ReRam) applications, a study has been conducted using embedded manganese oxysulfide (MOS) nanoparticles on the thin film of carbon nitride (CN). A high-temperature in-situ route was employed to synthesis CN-MOS composite where thiourea and manganese chloride was used as the precursor. The electrical property of the CN-MOS composite system (active layer), sandwiched between two gold electrodes, was measured under different sweeping (voltage) conditions. The device displayed different types of switching patterns, unipolar, and bipolar, by changing the sweep direction. The CN-MOS based device also exhibited good endurance and memory retention performances for the period of 10^4^ cycles and 10^4^ s, respectively, for both the polarities.

## Introduction

Resistive random access memory (ReRAM) devices with a simple metal–insulator–metal structure have received great attention due to its promising performance, structural simplicity, speed, outstanding endurance and low power consumption^1,2^. Apart from the memory behaviour, the devices have the potential to be applied in artificial intelligence and neuromorphic computing applications^3,4^. The ReRAM has been designated as one of the promising next generation non-volatile memories which functions between the high resistance state (HRS) and low resistance state (LRS) depending on the applied voltage^5,6^.

Metal oxide-based ReRAM devices have gained strong interest because of both economic and synthesis point of view. The device made with various metal oxides including TiO_2_^7^, HfO_2_^8^, ZnO^9^, Ta_2_O_5_^10^, Bi_2_O_3_^11^ and MnO^12^ have been exhibited non-volatile memory characteristics. Among the various metal oxides, manganese oxide is quite interesting as it has been used for thousands of years as pigments and glass cleaning agent and today as catalysts, battery and memory material.

Resistive switching property of manganese oxide and hafnium oxide based double-layer film exhibited bipolar resistive switching with a forming-free behavior. The double-layer film exhibited a high resistance ratio and good retention property as compared with the single layer of individual oxides^13^. Bilayer heterostructured device made with manganese oxide and indium-gallium-zinc oxide was reported for volatile and multistate nonvolatile resistive switching memory applications. The coexistence of volatile and nonvolatile switching characteristics was demonstrated by controlling the compliance current^14^. An improved resistive switching characteristic was demonstrated in a hybrid device of MnO (thin-film)-MnO (nanoparticle). The device exhibited stable unipolar switching behaviour with good endurance and retention characteristics. The conduction mechanisms in high resistive state was fitted with Schottky conduction and Poole–Frenkel emission whereas in low resistive state the Ohmic conduction behavior was dominated^12^. A ZnO-Mn based system sandwiched between silver and platinum electrodes exhibited ultrafast programming speed, high OFF–ON ratio with long retention time where redox-controlled Ag-bridge creation and rupture process was explained the existence memory effect of the device^15^. The resistive switching performance of MnO-based ReRAM was reported for nonvolatile resistance memory applications with Pt–Pt and Pt–Al electrode systems where the Pt–Al electrode system exhibited better endurance performance than the Pt–Pt based device. Both the devices displayed nonpolar resistance switching phenomenon by the formation and rupture of conductive filaments where the switching between HRS and the LRS was achieved under both DC-sweeping and pulse voltage conditions^16^. Manganese based intermetallic compound (Pr_0.7_Ca_0.3_MnO_3_) also showed electric-field-induced resistance switching where space-charge-limited-current and Poole–Frenkel were followed as the main conduction mechanism^17^.

In this paper, we investigate the resistive switching behaviour of manganese oxysulfide (MOS) nanoparticles, deposited on carbon nitride thin film. The composite system has been synthesized by applying a high temperature in-situ route. The CN-MOS composite system was used as an active material for resistive random access memory application. The endurance and nonvolatile behavior of the device were studied for 10^4^ cycles and for 10^4^ s, respectively. The resistive switching property of manganese oxysulfide based material has not been reported before and the material displayed the evidence for the sweep dependent transformation of polarity.

## Result and discussion

**Figure 1:**
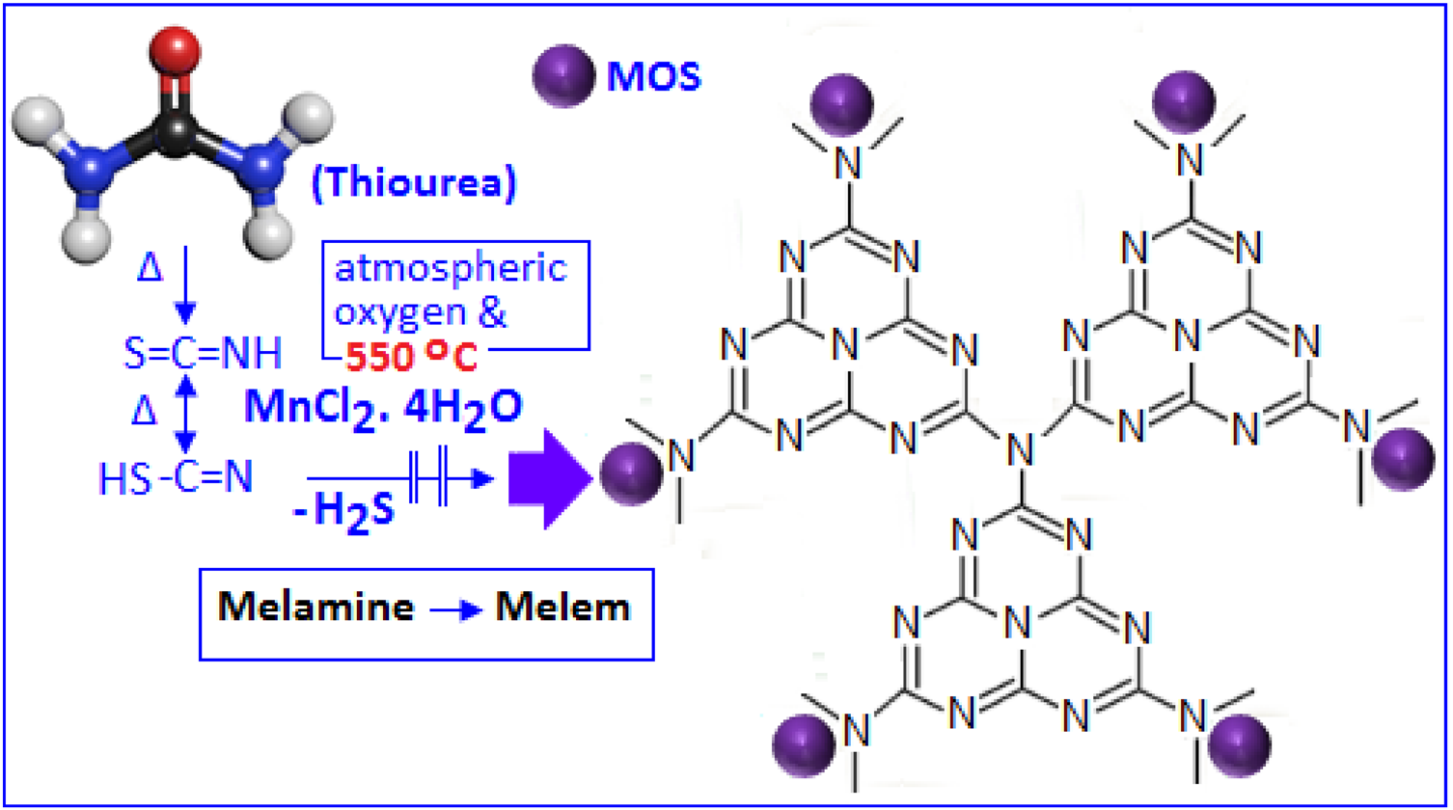
The formation mechanism of carbon nitride supported manganese oxysulfide (CN-MOS) hybrid system.

**Figure 2:**
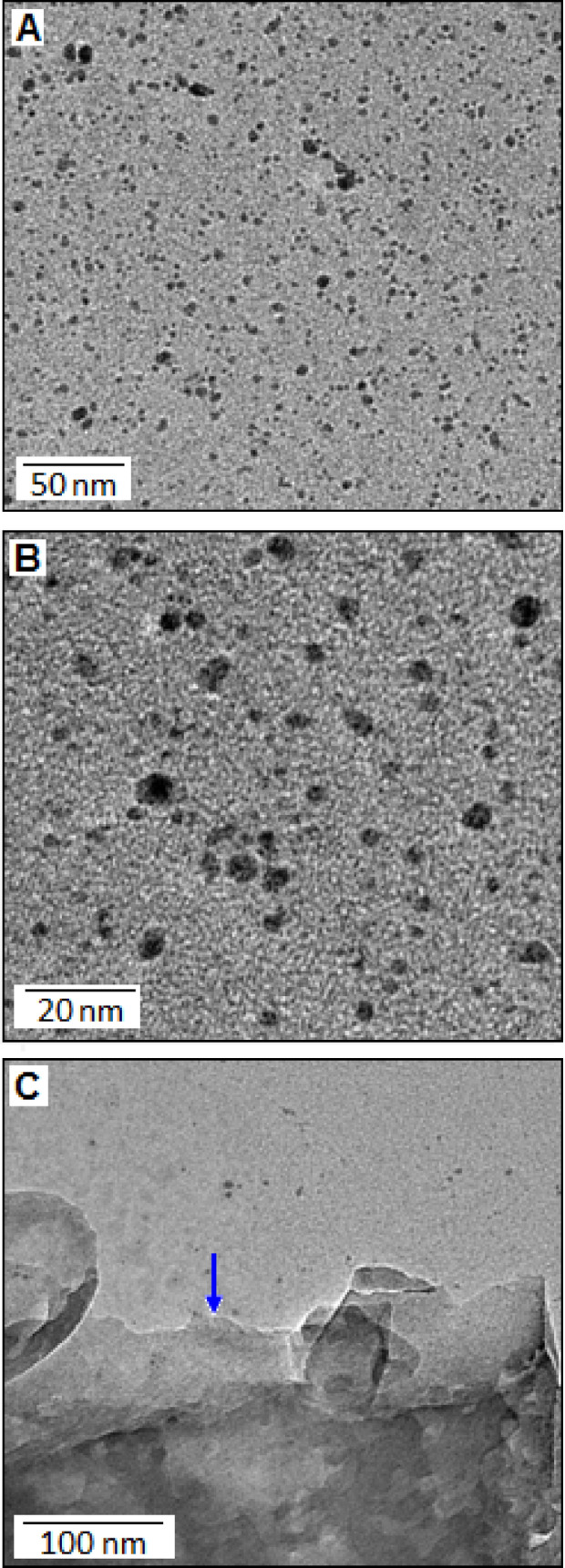
The TEM image of the manganese oxy-sulfide nanoparticles (black dots) with different magnifications (**A**,**B**). (**C**) The edge of a carbon nitride thin film (directed by a blue arrow).

**Figure 3:**
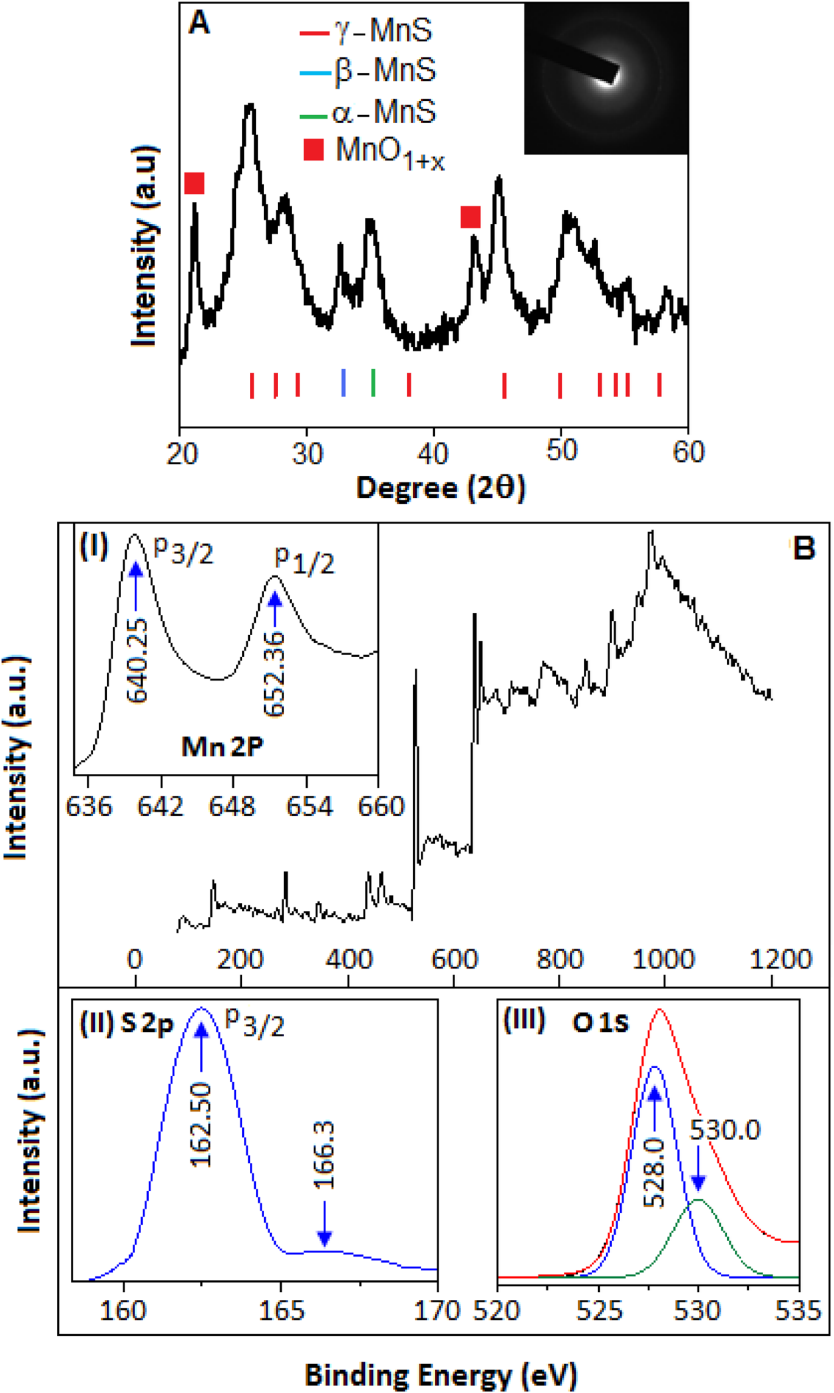
(**A**) X-ray diffraction pattern of manganese oxy-sulfide. The overall pattern comprises the mixed phases of MnS (α, β and γ) and off-stoichiometric manganese oxide (MnO_1+X_), red symbol, lattice structure. (**B**) The survey spectrum (XPS) of manganese oxy-sulfide (main-panel). In-set (I), (II) and (III) exhibit the core levels Mn 2p, S 2p and O1s spectra, respectively.

At the elevated temperature, thiourea finally converted to carbon nitride via different steps, such as, melamine and melem formation. The presence of the sulphur species in thiourea offered an extra chemical control for the synthesis of carbon nitride networks through polycondensation process^18^. During the reaction, hydrogen sulfide was evolved that reacted with manganese chloride in presence of atmospheric oxygen and formed manganese oxysulfide (MOS). The manganese oxysulfide, in the form of nanoparticles, were embedded on the carbon nitride networks. The schematic diagram (Fig. 1) shows the formation mechanism of CN-MOS composite system. The TEM image (Fig. 2A,B) shows highly distributed MOS nanoparticles (black dots) on the carbon nitride surface with different magnifications. The microscopic images also indicate a wide size distribution of the particles. Figure 2C specifies the edge of a thin film of carbon nitride (directed by a blue arrow). Figure 3A shows the XRD pattern of CN-MOS composite system recorded within the range (2θ) from 20° to 60°. The diffracted pattern revealed the presence of a mixed phases of hexagonal γ-MnS (JCPDS: 401289 space group: P63mc, red bar) with lattice constant a = b = 3.98 Å, c = 6.43 Å and cubic phases of β-MnS (JCPDS: 401288, blue bar) and α-MnS (JCPDS: 060518, green bar) with lattice constant of a = b = c = 5.61 Å and a = b = c = 5.22 Å, respectively, in the manganese oxysulfide structure. In addition to that, off-stoichiometric manganese oxide (MnO_1+X_) peak is also identified positioned at (21.1° and 43.1°, in red symbol) in the manganese oxysulfide structure. The existence of meta-stable manganese oxysulfide structure has already been reported with varying ratio of oxygen and sulphur content^19–21^. The broadening of the X-ray diffraction signatures indicate the amorphous nature of the nanoparticles, also supported by selected area diffraction pattern (Fig. 3A, in-set). The X-ray photoelectron spectroscopy technique was applied to determine the oxidation state of Mn, S and O. The survey spectra of the synthesized material is displayed in Fig. 3B (main panel). Figure 3B, in-set (I) and (II), showed the core levels Mn 2p and S 2p spectra, respectively. The Mn 2p_3/2_ and Mn 2p_1/2_ peaks were observed at 640.25 and 652.36 eV, respectively. The presence of S 2p_3/2_ peak at 162.2 eV attributed the sulfide anion^22^, whereas another high energy peak at 166.3 eV can be assigned for the presence of oxysulfide ion (oxidized sulfur)^22–24^. The O1s XPS spectra of the manganese oxysulfide sample is shown in Fig. 3B, inset (III). The XRD, XPS and TEM analysis confirmed the formation manganese oxysulfide nanoparticles on the layered structure of carbon nitride.

**Figure 4:**
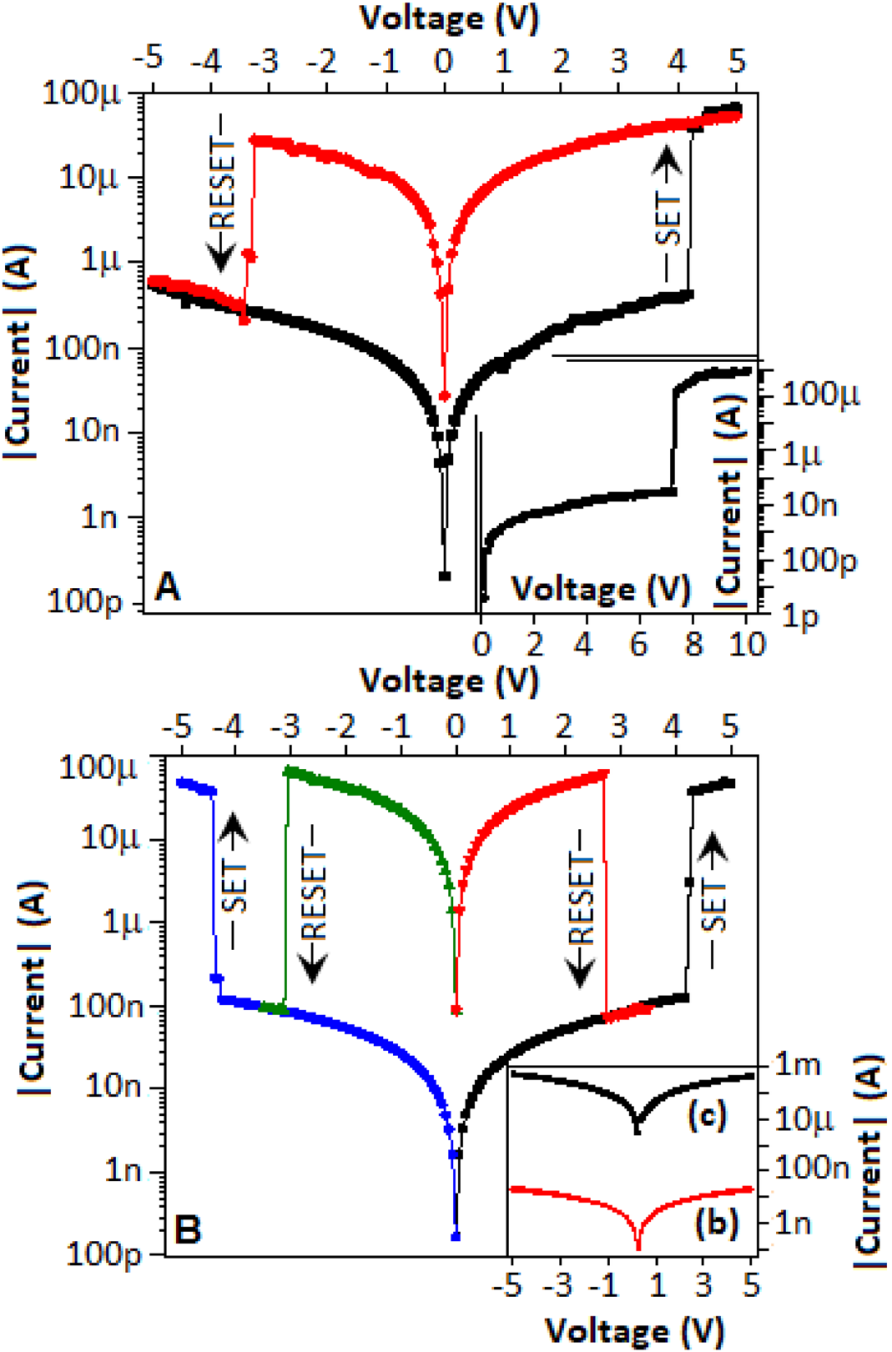
(**A**) Inset: The electroforming process of the fabricated device during the initial voltage sweep from 0 to + 10 V. The bipolar nature of the device exhibited during the voltage sweep from + 5 to − 5 V and from − 5 to + 5 V. (**B**) The unipolar nature of the device during the repeated voltage sweeps from 0 to + 5 and 0 to − 5 V. (**B**) Inset: I–V characteristics of CN-MOS (b) and CN-MOS (c) material based devices.

The fabricated device (Au║CN-MOS║Au), made with CN-MOS (a), was initially subjected to the voltage scan from 0 to + 10 V, where an electroforming process was observed at 7.2 V, which is energetically high enough for the energy requirement of conductive filament formation (Fig. 4A, inset). Further sweeping from + 5 to − 5 V, the device exhibited RESET behaviour (the process of transferring the device from high conductance state to low conductance state) at − 3.4 V. By reversing sweep direction from − 5 to + 5 V, the device displayed SET process at 4.3 V (the process of transferring the device from low conductance state to high conductance state). The current–voltage (I–V) graph (in semi-log), Fig. 4A, exhibits the bipolar in nature. Further, the I–V behaviour for the sweep direction from 0 to + 5 V exhibited the SET process at 4.1 V. By re-sweeping, for the same voltage range and direction, the device exhibited a RESET process at 2.7, an evidence of unipolar switching towards the positive voltage direction. Again, by applying a voltage sweep from 0 to − 5 V, the device exhibited SET at − 4.3. In a similar fashion by re-sweeping from 0 to − 5 V, the RESET process happened at − 3.1 V, an evidence of unipolar switching towards the negative voltage direction (Fig. 4B). The device exhibits bipolar and unipolar characteristics through reverse and re-sweeping of voltage, respectively.

For unipolar resistive switching (URS), the switching is induced by a voltage with the same polarity but SET and RESET occur in a different magnitude. For bipolar resistive switching (BRS), one polarity is required for SET and the opposite polarity is required for RESET. A third resistive-switching behavior can be categorized as nonpolar switching, in which the RESET process and the SET process are achieved for both positive and negative voltage polarity^25^. It is important to mention that all nonpolar are unipolar but all unipolar are essentially need not to be a nonpolar characteristics^25,26^. If a device exhibits both URS and BRS, it can be described as nonpolar^27^. In the current scenario, the SET/RESET was achieved by one type of voltage polarity and RESET/SET was obtained by reversing the polarity of the voltage, Fig. 4. The coexistence of both URS and BRS designated the device as a typical example of nonpolar characteristics^25–28^.

The device made with CN-MOS (c) exhibited higher conductance, Fig. 4B (inset), BLACK in colour, while the device made by CN-MOS (b), Fig. 4B (inset), RED in colour, exhibited low conductance where both the devices exhibited no resistive switching effect.

The coexistence of BRS and URS modes has been observed in Ag-Zn_0.98_Cu_0.02_O-ITO based device structure where the formation and rupture of conducting filaments, due to the creation of oxygen vacancies, are responsible for the presence of both BRS and URS modes in the device^29^. Again, depending on the compliance current during the electroforming process, either BRS or URS was observed in Pt–TiO_2_–Pt based device. With a lower compliance current (0.1 mA) during electroforming, asymmetric current–voltage curves exhibited BRS, while with a higher compliance current (1–10 mA) URS behavior was observed. The permanent transition from BRS to stable URS was attained by applying a voltage with a compliance current of 3 mA^30^. A recent report showed both BRS and URS behaviors for the Ag-Ti/CeO_2_-Pt based device, where the switching effect in BRS was dominated by the electrochemical metallization mechanism, while in URS, the conduction property was dominated by thermochemical mechanism^31^. Both BRS and URS behaviors were observed in Cu_2_O/Ga_2_O_3_ layer based device and the conduction mechanism of the two switching modes are due to the combined action of the trapping and detrapping effects and the migration of oxygen vacancies at the interface between the layers^32^. The coexistence of the BRS and URS modes for the Ni–NiO–Ni based memory device has been reported where the URS was associated with thermal-based mechanism, while the voltage-controlled electrochemical reaction was responsible for the BRS^33^. More examples regarding the coexistence of BRS and URS modes and other parameters for the ReRAM devices are available elsewhere in the manuscript as a ready reference for the readership, table T1, supplementary information.

**Figure 5:**
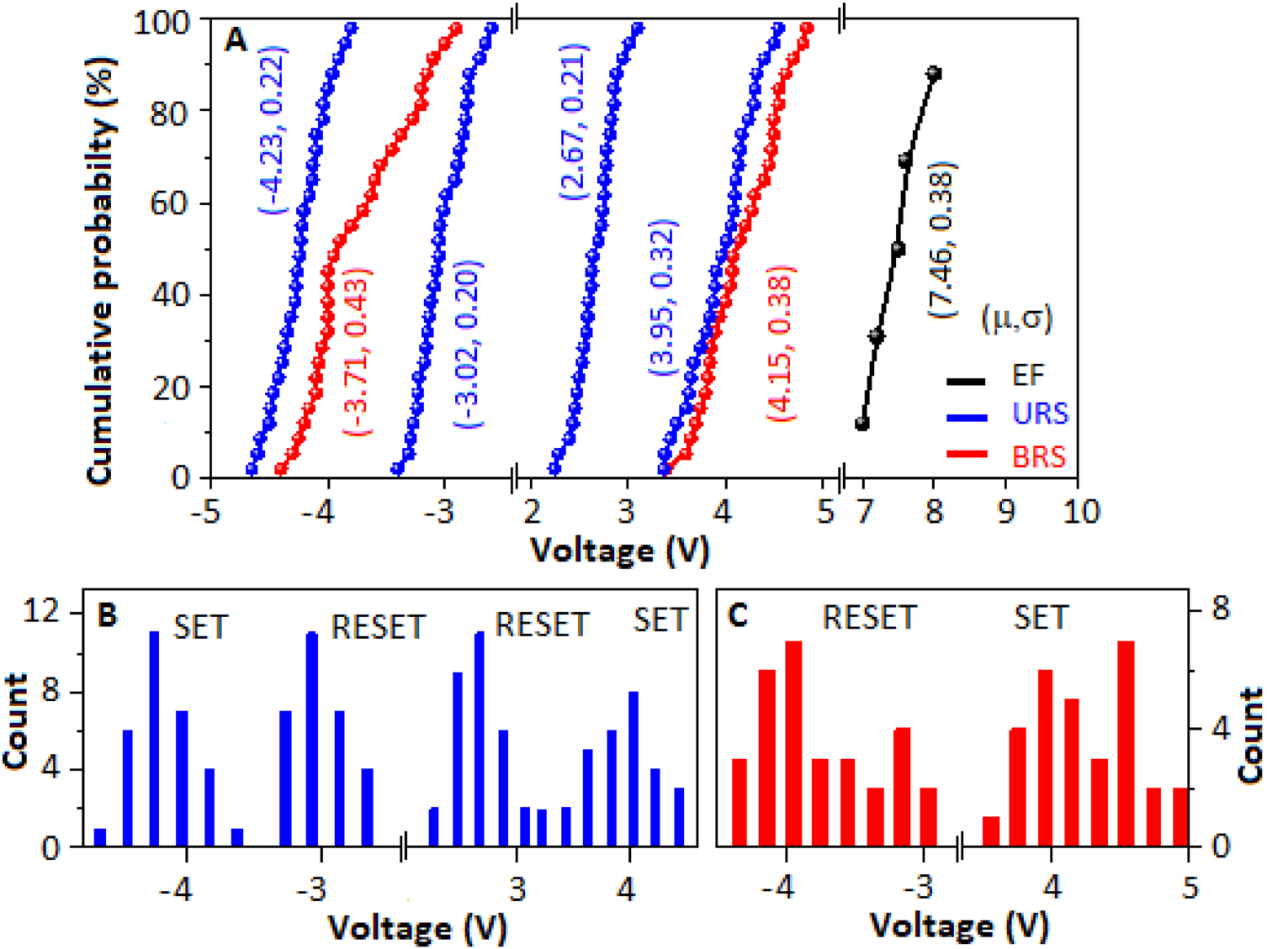
(**A**) The cumulative distribution of the operating SET, RESET and electroforming voltage of the CN-MOS film-based ReRAM device. (**B**) and (**C**) display the distribution of the SET and RESET voltages in successive cycles for URS and BRS, respectively.

The I–V characteristics of five more devices (D1-D5), made with CN-MOS (a), are incorporated in the supplementary information, figure S1, where all the devices exhibited the sweep dependent change of polarity (BRS and URS). The cumulative distribution of the operating SET, RESET and electroforming voltage of the CN-MOS (a) based ReRAM devices are exhibited in Fig. 5. The mean (µ) and standard deviation (σ) values of SET and RESET voltages, for both BRS and URS, are mentioned in Fig. 5A (and also in tabular form, table T2, supplementary information). Figure 5B,C, exhibited the distribution of the SET and RESET voltages in successive cycles for URS and BRS, respectively, for the CN-MOS (a) based ReRAM devices (D1–D5). The distribution results imply that the URS phenomenon is more stable under electrical stress condition than BRS, as the density of states are more concentrated in URS.

### Mechanism of the oxygen-deficient filament formation and dissolution

The filament formation within the device made with manganese oxysulfide (active material) can be explained by the formation of oxygen ion vacancy, as conductive channel^34^. A positive voltage (from 0 to + 10 V) on the electrode attracts oxygen ions from the top interface of the active material and repels oxygen vacancies to the bottom interface. The oxidation of the oxygen ions leads to oxygen gas eruption and the formation of oxygen vacancy. The continuous redox reaction further leads to migration of oxygen vacancies to the bottom interface, which ultimately form the oxygen vacancy filament and the device switched to ON-state.

### Mechanism for the bipolar behaviour of the device

An applied potential (from + 5 to − 5 V) drove the oxygen ions away from the top interface and oxygen vacancies departed from the bottom interface. At − 3.4 V, a disintegration of the oxygen-deficient filament pushed back the device to the OFF-state, Fig. 4A. During the filament formation and rapture process, the oxygen distribution has been partially reoriented from the lattice site to the non-lattice site (at the interface of CN) of the manganese oxysulfide, where the oxygen species was loosely bounded. It is documented in the literature that the involvement of non-lattice oxygen plays a crucial role in the resistive switching^35–38^. For an applied potential from − 5 to + 5 V, the device transformed from OFF state to ON state at 4.3 V, Fig. 4A, due to the creation of facile oxygen vacancy at the non-lattice site.

### Mechanism for the unipolar behaviour of the device

When the voltage was applied from 0 to + 5 V, the device exhibited a transformation from OFF state to ON state at 4.1 V also due to the above mentioned oxygen vacancy formation mechanism at the non-lattice site. The evolved oxygen gas was adsorbed by CN, as the nitrogen atoms support to increase the electropositive character of adjacent carbon atoms, which might reduce the energy barriers of oxygen adsorption^39,40^. A subsequent voltage sweep from 0 to + 5 V, exhibited the device still in high conductance state due to the previously formed oxygen vacancies at the non-lattice site. At the voltage 2.70 V, the device transformed from ON state to OFF state due to desorption and subsequent occupation of oxygen from the CN to the non-lattice site of manganese oxysulfide, respectively. Compared to lattice oxygen, the non-lattice oxygen can restore the HRS more easily by removing the oxygen vacancies generated during the SET process^41^.

### Carrier transport mechanism

**Figure 6:**
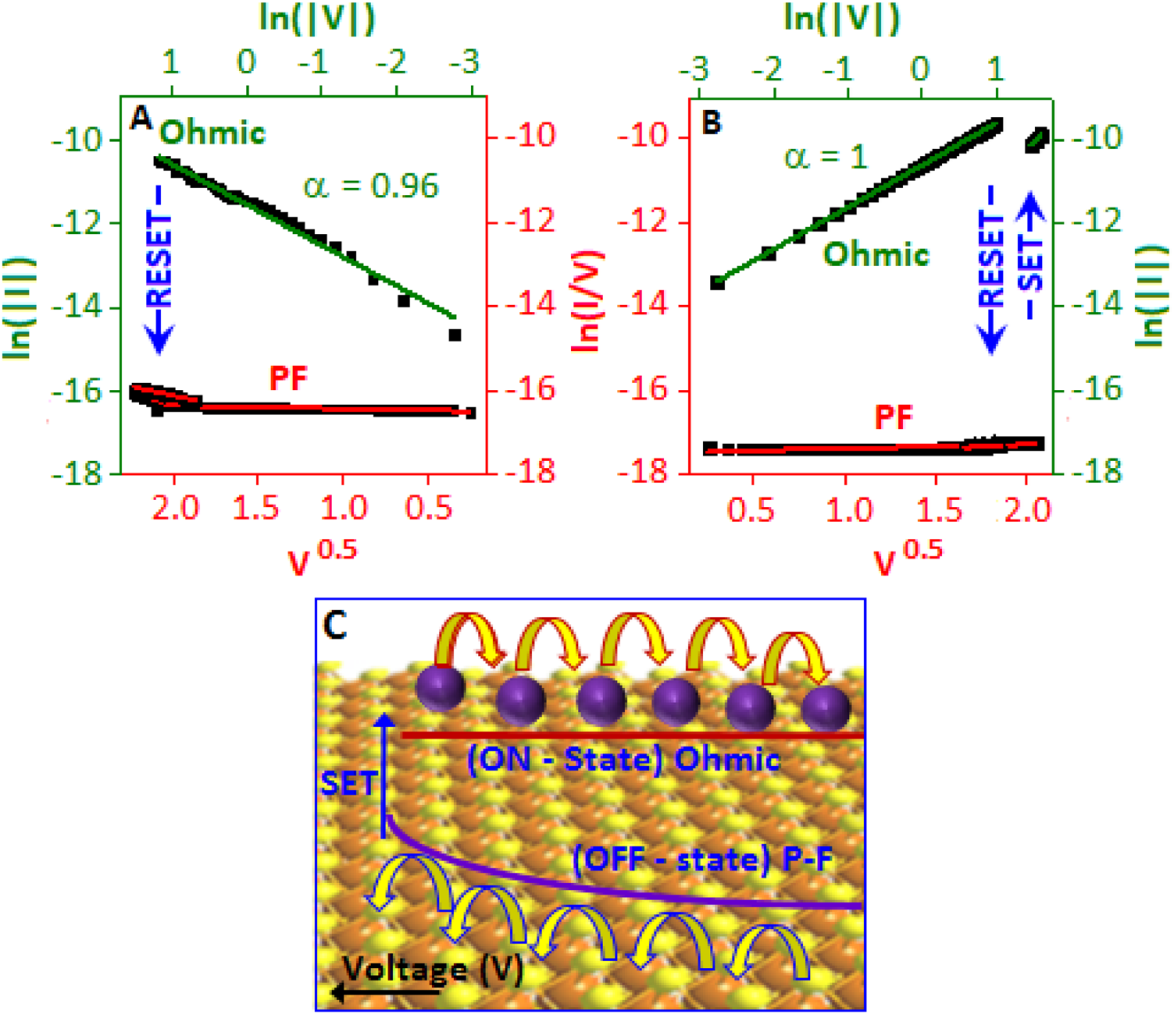
The current–Voltage behaviour of the bipolar and unipolar events fitted with the P–F and Ohmic for OFF-state and ON-state, respectively. The fitting was performed based on the (**A**) negative side of the bipolar and (**B**) positive side of the unipolar, switching events. (**C**) Schematic demonstration of the charge transport mechanism.

The carrier transport mechanism is graphically represented in Fig. 6A,B and schematically demonstrated in Fig. 6C. Initially, the electrons are trapped in the localized states of the valence band of carbon nitride (OFF-state). Due to the gradual increase of voltage, the carriers exit from its localized state and passed through the higher density of states. At this stage, the electron migration mechanism was followed by Poole–Frenkel (PF) emission, ln (I/V) vs. V^0.5^. A field induced band bending effect^42^ reduced the band gap between the valence band and conduction band. At a particular threshold voltage, the electrons migration took place from the valence band of CN to the conduction band of manganese oxysulfide particles through the conduction band of CN. The transport mechanism in the conduction band of manganese oxysulfide was followed the Ohmic behaviour and fitted with ln (I) vs ln (V), ON-state. The above mechanism is valid for the both sides of both unipolar and bipolar systems for all ON-states and OFF-states. The device behaviour for both CN-MOS (b) and (c) can be explained by the amount of manganese oxy-sulfide particle formation. The higher conductance indicates nanoparticles played the major role for the conduction process, whereas low conductance suggests that carbon nitride dominated the charge transport process.

### Endurance behaviour of the device

**Figure 7:**
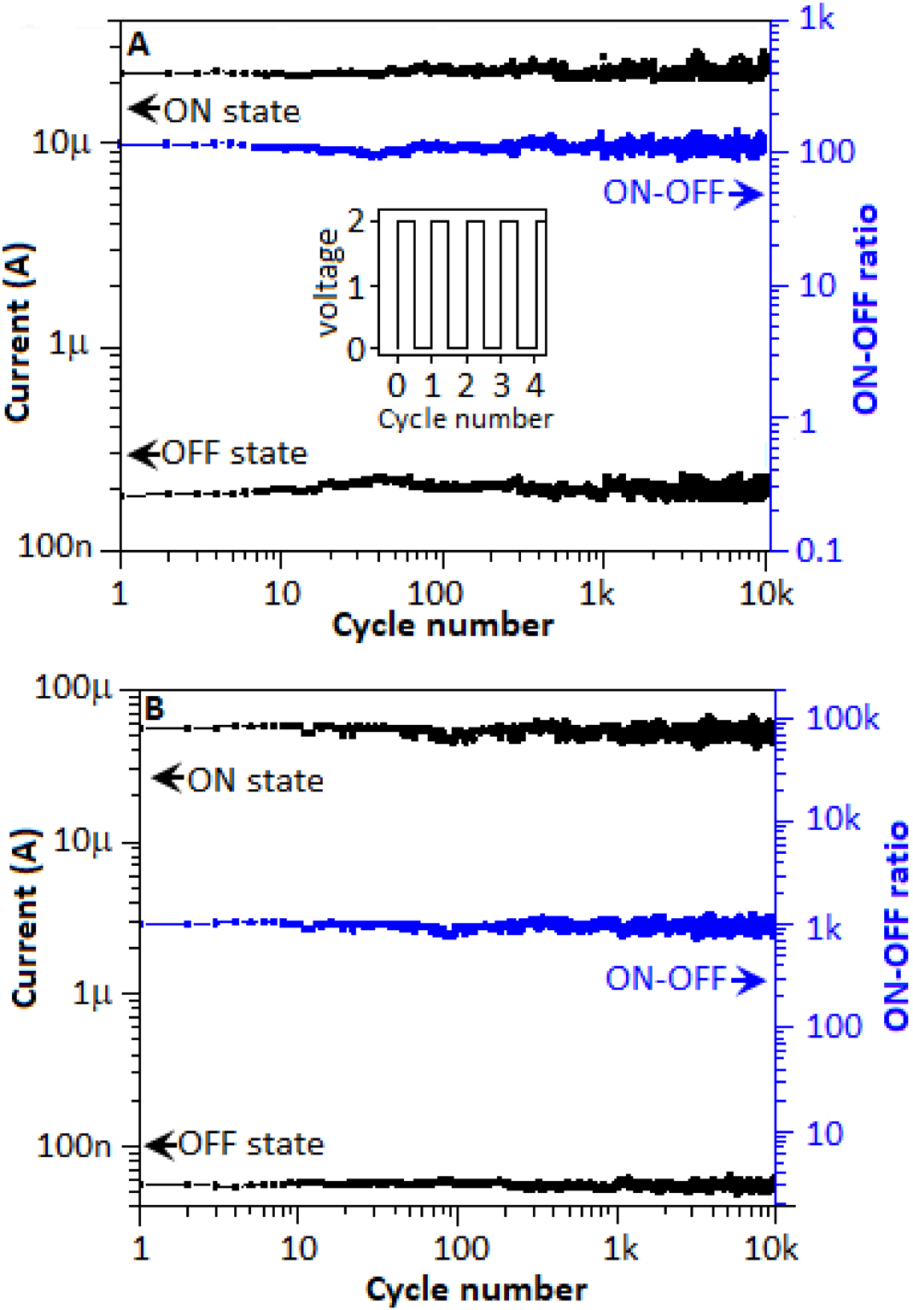
The endurance study of the fabricated device for (**A**) bipolar and (**B**) unipolar events. (**A**), inset, 2 V pulse train for 10^4^ cycles assigned for the study.

The endurance behaviour is one of the important properties of a memory device for its practical application. In the current experiment, a pulse train for 10^4^ cycles, where each pulse consist of 0.1 s of 2 V for every 0.1 s time interval (Fig. 7A, inset), was applied to the device. For the bipolar behaviour (Fig. 7A) of the device, voltage scan from − 5 to + 5 V (ON-state) and from + 5 to − 5 V (OFF-state), the above mentioned pulse train was applied to the device to perform the endurance study. In the case of unipolar endurance study (Fig. 7B), the device was initially scanned from 0 to + 5 V and after attaining the ON-state (at 4.1 V) the pulse train was applied to survey the ON-state endurance characteristic. Again, the device was scanned from 0 to + 5 V and reached to the OFF-state position at 2.7 V and the similar pulse train was applied to survey the OFF-state endurance. Both the ON- and OFF-state endurance behavior of the device for bi- and unipolar characteristics are plotted in Fig. 7A,B, respectively. Low fluctuations of the current values were observed during the entire cycling process for both low and high conductance states, which indicates the device had a good endurance property.

### Retention study of the device

**Figure 8:**
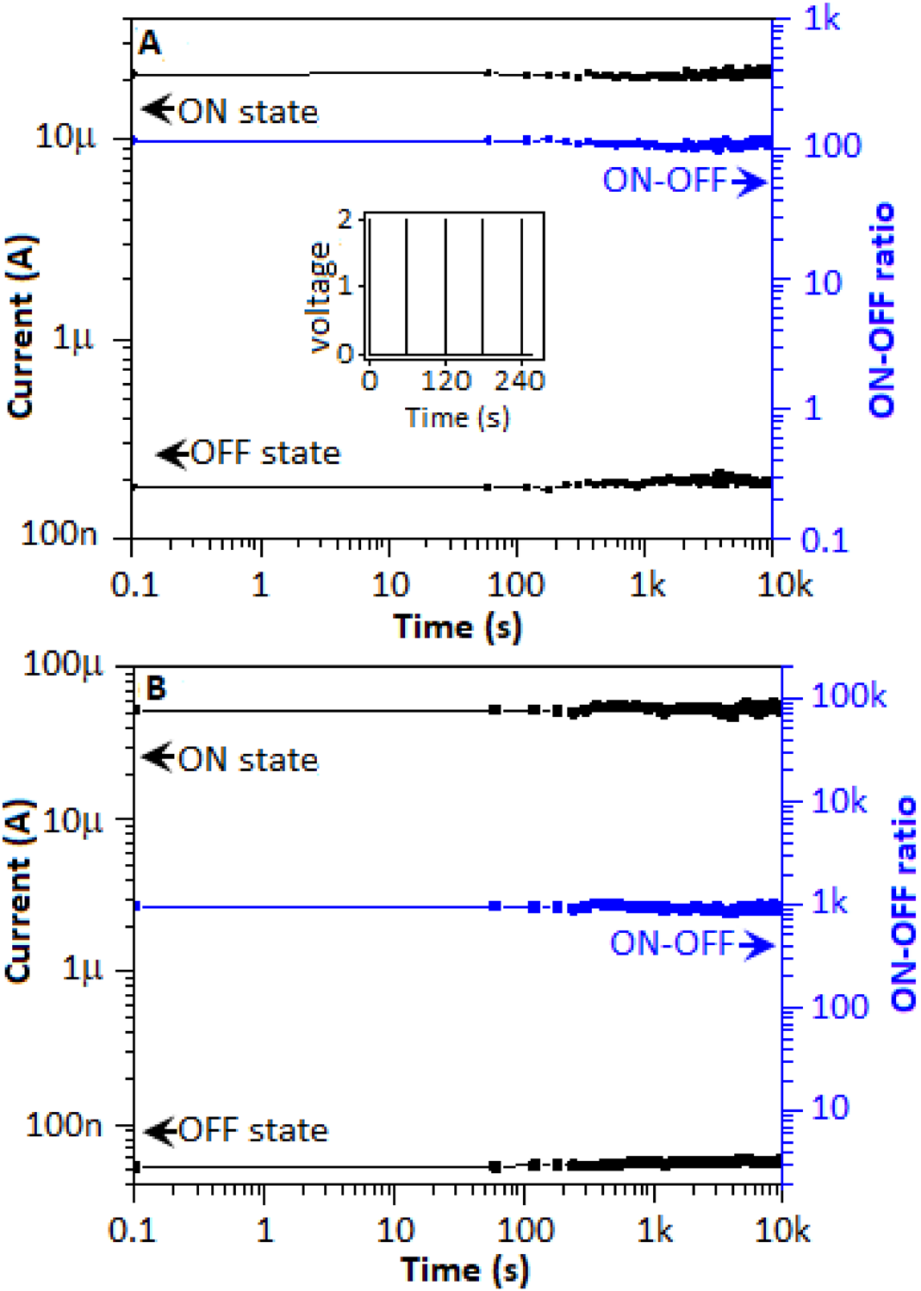
The nonvolatile property of the device for (**A**) bipolar and (**B**) unipolar events. (**A**) inset, 2 V pulse train for 10^4^ s assigned for the study.

Retention study indicated the ability of the device to maintain its conductance state irrespective of refreshing power supply, an important characteristic of the memristor. For the retention study, a pulse train for 10^4^ s was applied to the device where each pulse consist of 2 V for 0.1 s after every 60 s (Fig. 8A, inset). A similar approach, as endurance study, was applied for retention study to analyze the ON-state and OFF-state for the bipolar and unipolar systems of the device and plotted in the Fig. 8A,B, respectively. The study endorsed an excellent retention property of the device.

**Figure 9:**
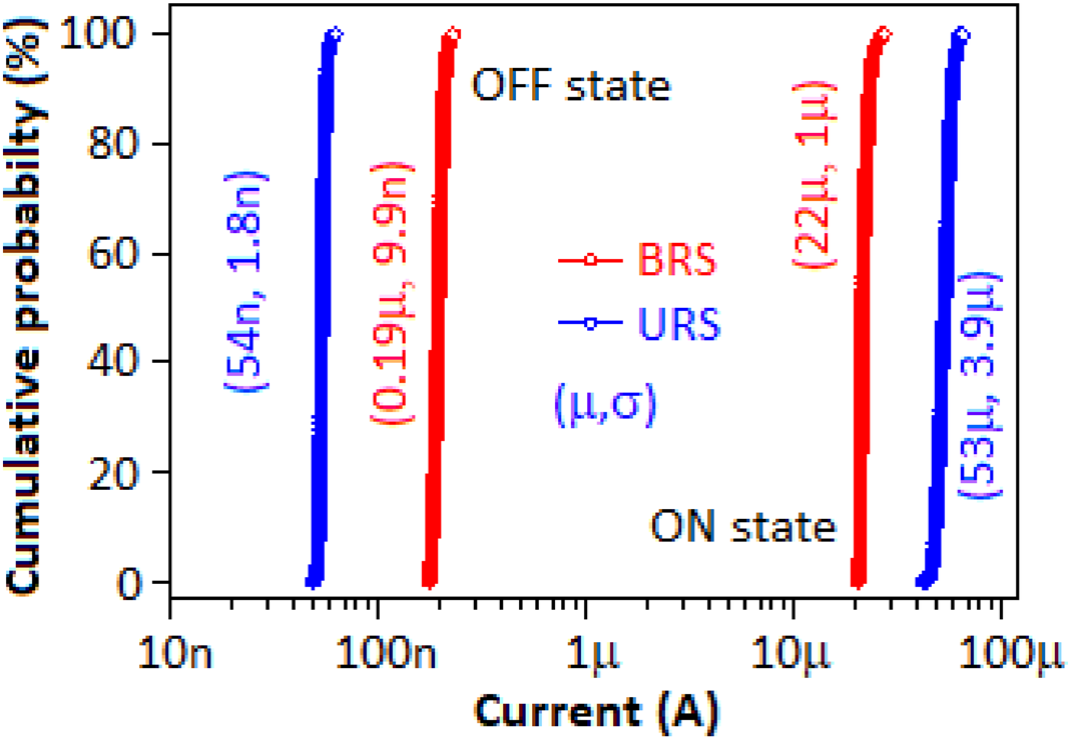
The cumulative distribution of the OFF and ON state currents for BRS and URS of the device. The mean (µ) and standard deviation (σ) of the OFF and ON state currents for BRS and URS are incorporated in the figure.

The above two properties (endurance and retention) of the device exhibited a consistent ON–OFF ratio with the values of 10^2^ and 10^3^ for both bipolar and unipolar systems, respectively. The cumulative distribution of the OFF and ON state current for BRS and URS of the CN-MOS (a) based ReRAM device is displayed in Fig. 9 with the mean (µ) and standard deviation (σ) values, based on the endurance study (Fig. 7A,B).

## Conclusion

The report described the electrical property of a device, made with the hybrid system of manganous oxysulfide nanoparticles and carbon nitride, which exhibited sweep dependent varied resistive switching behavior. The device initially went through a filament formation pathway through the creation of oxygen vacancy and after that exhibited bipolar and unipolar switching events, depending on the applied sweep directions. The device was tested for endurance property with a pulse train of 10^4^ cycles. For both the ON- and OFF-state, the endurance behavior of the device for bi- and unipolar events showed minimum fluctuations of the current values during the entire cycling process. The hybrid device was also exposed for retention study with a pulse train of 10^4^ s to analyze the ON-state and OFF-state for both bipolar and unipolar occasions. The endurance and nonvolatile studies exhibited a consistent ON–OFF ratio with the values of 10^2^ and 10^3^ for bipolar and unipolar systems, respectively. The charge transport mechanism was followed with Poole–Frenkel (PF) emission for the OFF-state and Ohmic behaviour for the ON-state. The results are encouraging for the practical use of nanoparticles of manganous oxysulfide based materials for the nonvolatile resistive memory application with the scope of miniaturization of the device size.

## Methods

### Synthesis of CN-MOS composite system

For the synthesis of CN-MOS composite system, manganese chloride and thiourea (1:5, by weight) were thoroughly mixed. The homogeneous blend was transferred into the quartz made reaction chamber with a hole and finally inserted in a programmable furnace (with the heating rate of 5 °C. min^−1^ for 550 °C and cooled down at room temperature). At the end, the material, CN-MOS (a), was collected from the reaction chamber and characterized for microscopic, structural and electrical properties. Two other samples, CN-MOS (b) and CN-MOS (c) were prepared under the identical reaction conditions, as above, using the manganese chloride and thiourea composition ratio 0.5:5 and 2:5 (by weight), respectively.

### Device fabrication

The device was fabricated by applying the previously reported method^43^. The gold electrode was printed on a paper substrate and the CN-MOS (a) material was deposited on the top of the printed electrode using a spin-coating technique by applying a solution processing method with the thickness of approximately 200 nm. Finally, the top electrode was fabricated with the gold (active area of 0.5 × 0.5 mm^2^) and the current–voltage measurement was performed with the fabricated device (Au║CN-MOS║Au), shown in figure S2.

## Supplementary information

Supplementary file1
