# Supplementary material for: Resistive switching characteristics of carbon nitride supported manganese oxysulfide: an evidence for the sweep dependent transformation of polarity
Source: Sci Rep. 2020 Aug 31;10:14319. doi: 10.1038/s41598-020-71313-2 (PMC7459303; doi:10.1038/s41598-020-71313-2)
Supplement: Supplementary file 1 — Supplementary file1 [file 41598_2020_71313_MOESM1_ESM.docx]

**Supplementary information**

Resistive switching characteristics of carbon nitride supported manganese oxysulfide: An evidence for the sweep dependent transformation of polarity

Venkata K Perla, Sarit K Ghosh, Kaushik Mallick*

Department of Chemical Sciences, University of Johannesburg, P.O. Box: 524, Auckland Park, 2006, South Africa. Correspondence and requests for materials should be addressed to K.M. (e-mail: kaushikm@uj.ac.za)


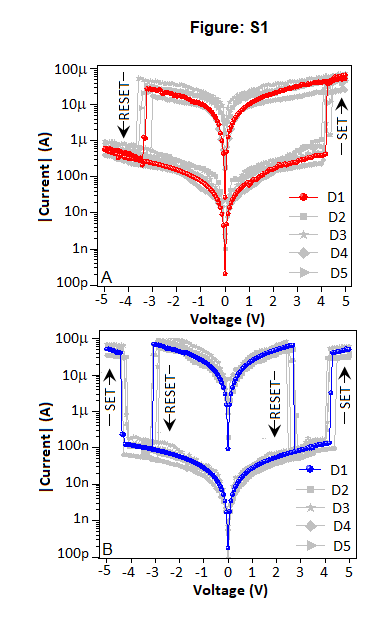


**Figure S1:** The devices (D1-D5) exhibited (A) the bipolar nature during the voltage sweep from +5 to -5V and from -5 to +5V, and (B) the unipolar nature during the repeated voltage sweeps from 0 to +5 V and 0 to -5 V.


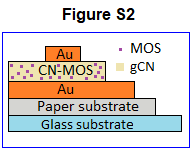


**Figure S2:** The schematic cross section view of the device (Au║CN-MOS║Au)

**Table T1:** A literature review for the ReRAM devices with both BRS and URS characteristics and other properties (device structure, ON-OFF ratio, endurance and retention) are summarized below.

| Entry | Material (M) | Device structure | ON-OFF ratio BRS (URS); Endurance BRS (URS); Retention BRS (URS) | Mechanism of transition between BRS and URS |
| --- | --- | --- | --- | --- |
| 1 | TiO_2_ | Pt-M-Pt | 5 (10^3^); 30(80); NA | Icc dependent electroforming process |
| 2 | NiO | Ni-M-Ni/TiN/Ti/SiO_2_ /Si_3_N_4_ /Si | 10^2^(10^2^); 10^2^(10^2^); NA | Driven by electrochemical and thermochemical process |
| 3 | MoS_2_ | Ag-M-Au/Ti/PET | 2×10^1^(2×10^1^); 10^4^(10^4^); 10^4^(10^4^) | Sliver and sulphur vacancies are responsible |
| 4 | Cu_2_O/Ga_2_O_3_ | Au/Ti-M-ITO | 10^2^(10^2^); 10^2^(10^2^); 2×10^2^(3×10^2^) | The oxygen vacancies formation at the interface between the active material along with the bias control filament formation and depletion |
| 5 | Ti/CeO_2_ | Ag-M-Pt | <10^2^(<10^2^); NA; NA | Driven by electrochemical and thermochemical process |
| 6 | Zn_0.98_Cu_0.02_O | Ag-M-ITO | 10^6^(10^4^); NA; 10^3^(10^3^) | Formation of O-vacancies at the interface |
| 7 | Au-GO | ITO-M-Al | 2×10^2^(5×10^2^); 1×10^2^ (1×10^2^); 4×10^3^(4×10^3^) | Driven by device geometry and device processing |
| 8 | BFMO | Pt-M-Pt | 10(10); 2×10^2^ (1×10^2^); NA | The valence fluctuation of the active material and the O-vacancy formation |
| 9 | GaOx/NiOx | Au/Ti-M-ITO | 5(10); 10^2^(10^2^); 10^2^(10^2^) | Schottky junction formation and interfacial variation of the active materials |
| 10 | ZnO | Ag-M-Pt | 10^2^(10^2^); 10^2^(2×10^2^); NA | Directed by Joule-heating and electrochemical redox reaction |
| 11 | Ag-NiFe_2_O_4_ | Pt-M-Pt/Ti/SiO_2_/Si | 10^2^(10^3^); >10^2^ (10^3^); 10^5^(10^5^) | Directed by Joule-heating and electrochemical redox reaction |
| Present work | CN-MOS | Au-M-Au | 10^2^ (10^3^); 10^4^ (10^4^); 10^4^ (10^4^) | Details are mentioned in the manuscript |

Compliance current (Icc); Bipolar resistive switching (BRS); Unipolar resistive switching (URS); graphene oxide (GO); Mn-doped BiFeO_3_ (BFMO)

**References:**

1. Jeong, D. S., Schroeder, H. & Waser, R. Coexistence of Bipolar and Unipolar Resistive Switching Behaviors in a Pt∕TiO2∕Pt Stack. *Electrochem. Solid-State Lett.*, **10**, G51, (2007).

2. Goux, L. *et al.* Coexistence of the bipolar and unipolar resistive-switching modes in NiO cells made by thermal oxidation of Ni layers. *J. Appl. Phys.*, **107**, 024512, (2010).

3. Zhao, X. *et al.* Reversible alternation between bipolar and unipolar resistive switching in Ag/MoS2/Au structure for multilevel flexible memory. *J. Mater. Chem. C*, **6**, 7195-7200, (2018).

4. Zhi, Y. S. *et al.* Reversible transition between bipolar and unipolar resistive switching in Cu2O/Ga2O3 binary oxide stacked layer. *AIP Adv.*, **6**, 015215, (2016).

5. Wang, W., Zhang, B. & Zhao, H. Forming-free bipolar and unipolar resistive switching behaviors with low operating voltage in Ag/Ti/CeO2/Pt devices. *Results Phys.*, **16**, 103001, (2020).

6. Xu, Q., Wen, Z. & Wu, D. Bipolar and unipolar resistive switching in Zn0.98Cu0.02O films. *J. Phys. D: Appl. Phys.*, **44**, 335104, (2011).

7. Khurana, G. *et al.* Non-Polar and Complementary Resistive Switching Characteristics in Graphene Oxide devices with Gold Nanoparticles: Diverse Approach for Device Fabrication. *Sci. Rep.*, **9**, 15103, (2019).

8. Luo, J. M., Lin, S. P., Zheng, Y. & Wang, B. Nonpolar resistive switching in Mn-doped BiFeO3 thin films by chemical solution deposition. *Appl. Phys. Lett.*, **101**, 062902, (2012).

9. Chu, X. L. *et al.* Interface induced transition from bipolar resistive switching to unipolar resistive switching in Au/Ti/GaOx/NiOx/ITO structures. *RSC Adv.*, **5**, 82403-82408, (2015).

10. Lee, S., Kim, H., Park, J. & Yong, K. Coexistence of unipolar and bipolar resistive switching characteristics in ZnO thin films. *J. Appl. Phys.*, **108**, 076101, (2010).

11. Hao, A. *et al.* Coexistence of unipolar and bipolar resistive switching behaviors in NiFe2O4 thin film devices by doping Ag nanoparticles. *J. Appl. Phys.*, **123**, 085108, (2018).

**Table T2:** The parameter µ (mean) and σ (standard deviation) values of the cumulative distribution for the SET, RESET and electro-forming voltages during the BRS and URS.

| **Parameter** | **μ (Mean) (V)** | **σ (Standard Deviation) (V)** |
| --- | --- | --- |
| Electroforming | 7.46 | 0.38 |
| BRS SET (RESET) | 4.15 (-3.71) | 0.38 (0.43) |
| URS SET (RESET) |  |  |
| Negative side | -4.23 (-3.02) | 0.22 (0.20) |
| Positive side | 3.95 (2.67) | 0.32 (0.21) |
